# Detection of Gastrointestinal Pathogens with Zoonotic Potential in Horses Used in Free-Riding Activities during a Countrywide Study in Greece

**DOI:** 10.3390/ani14172566

**Published:** 2024-09-03

**Authors:** Panagiota Tyrnenopoulou, Katerina Tsilipounidaki, Zoi Florou, Christos-Georgios Gkountinoudis, Konstantina Tyropoli, Alexandros Starras, Christina Peleki, Danai Marneris, Nikoletta Arseniou, Daphne T. Lianou, Eleni I. Katsarou, Efthymia Petinaki, George C. Fthenakis

**Affiliations:** 1 Veterinary Faculty, University of Thessaly, 43100 Karditsa, Greece; 2 University Hospital of Larissa, 41110 Larissa, Greece; 3 Private Veterinary Practice, 81107 Kalloni, Lesvos, Greece; 4 Private Veterinary Practice, 73100 Chania, Crete, Greece; 5 Private Veterinary Practice, 40200 Livadi, Elassona, Greece; 6 Private Veterinary Practice, 15450 Neo Psychico, Attica, Greece; 7 Private Veterinary Practice, 15340 Aghia Paraskevi, Attica, Greece

**Keywords:** *Campylobacter*, *Cryptosporidium*, diarrhoea, donkey, *Escherichia coli*, FilmArray, *Giardia*, horse, Norovirus, *Salmonella*, *Vibrio*, *Yersinia*, zoonotic infection

## Abstract

**Simple Summary:**

An extensive countrywide investigation was carried out in Greece to study gastrointestinal zoonotic pathogens in horses. Faecal samples were collected and tested. Gastrointestinal pathogens were detected in the faecal samples obtained from 43% of horses examined. They were detected more frequently in samples from horses in courtyard housing. The most frequently detected zoonotic pathogens were enteropathogenic *Escherichia coli* and Shiga-like toxin-producing *E. coli* stx1/stx2. The decreasing age of horses and the presence of livestock at the same premises as the horses emerged to be significantly associated with the detection of pathogens. The findings have indicated the presence of pathogens with zoonotic potential in horses and have suggested factors that may play some role in this.

**Abstract:**

The objectives of this study were (a) to detect zoonotic gastrointestinal pathogens in faecal samples of horses using the FilmArray^®^ GI Panel and (b) to identify variables potentially associated with their presence. Faecal samples collected from 224 horses obtained during a countrywide study in Greece were tested by means of the BioFire^®^ FilmArray^®^ Gastrointestinal (GI) Panel, which uses multiplex-PCR technology for the detection of 22 pathogens. Gastrointestinal pathogens were detected in the faecal samples obtained from 97 horses (43.3%). Zoonotic pathogens were detected more frequently in samples from horses in courtyard housing (56.0%) than in samples from horses in other housing types (39.7%) (*p* = 0.040). The most frequently detected zoonotic pathogens were enteropathogenic *Escherichia coli* (19.2% of horses) and Shiga-like toxin-producing *E. coli* stx1/stx2 (13.8%). During multivariable analysis, two variables emerged as significant predictors for the outcome ‘detection of at least one zoonotic pathogen in the faecal sample from an animal’: (a) the decreasing age of horses (*p* = 0.0001) and (b) the presence of livestock at the same premises as the horses (*p* = 0.013). As a significant predictor for the outcome ‘detection of two zoonotic pathogens concurrently in the faecal sample from an animal’, only the season of sampling of animals (autumn) emerged as significant in the multivariable analysis (*p* = 0.049). The results indicated a diversity of gastrointestinal pathogens with zoonotic potential in horses and provided evidence for predictors for the infections; also, they can serve to inform horse owners and handlers regarding the possible risk of transmission of pathogens with zoonotic potential. In addition, our findings highlight the importance of continuous surveillance for zoonotic pathogens in domestic animals.

## 1. Introduction

In horses, infectious diseases can incur substantial societal, industrial, and emotional costs, influencing event cancellations, import restrictions, and the psychological well-being of horse owners. Further, equine infections may also become significant in cases involving zoonotic pathogens, due to the frequent and close interactions between these animals and humans within the various equestrian settings [1,2,3]. The impact of infectious diseases in horses can be challenging, due to the absence of extensive surveillance systems like those in place for livestock and the availability of limited relevant data, where most information are derived from outbreak investigations and small-scale studies [4,5]. Estimates of the frequency of equine infections are often based on specific outbreaks or retrospective analyses, complicating assessment of their broader impact. Nevertheless, economic and societal consequences can be significant [6].

Gastrointestinal pathogens (viruses, bacteria, protozoa or metazoan) can cause significant health issues in horses [1,7,8]. The diagnosis of these infections is challenging due to the non-specific nature of clinical signs, which are often similar across different causal agents [9,10]. While detailed laboratory examinations of faecal samples can provide a definitive diagnosis, these tests can be time-consuming and may delay the initiation of appropriate treatment [11]. Moreover, various commonly employed diagnostic techniques have limited accuracy [12,13,14]. Therefore, there is a need for rapid and reliable diagnostic methods, for use in the diagnosis of equine gastrointestinal infections, which would enable timely interventions to mitigate the spread of zoonotic diseases.

A recent (July 2024) literature search in the Web of Science database by using the string [horse* OR *equine* OR *equus caballus*] AND [gastrointesti* AND zoono*] revealed a total of 17 relevant articles. These reported the isolation of *Cryptosporidium* spp. and *Giardia* spp. [15], *Escherichia coli* [16], *Helicobacter pylori* [17] and *Salmonella* spp. [18] from faecal samples of clinically healthy horses.

The ‘BioFire^®^ FilmArray^®^ Gastrointestinal (GI) Panel’ (BioFire Diagnostics, Salt Lake City, UT, USA) is a multiplex nucleic acid-based test, which is fully automated and can be employed for the qualitative in vitro detection and identification of multiple pathogens. The panel can simultaneously detect 22 different gastrointestinal pathogens (13 bacteria, 4 protozoa, and 5 viruses) and was developed primarily for the rapid detection and identification of a multitude of pathogens in clinical samples from people [19]. However, the panel was found to have high accuracy for detecting pathogens in other matrixes as well, for example, in water samples [20]. In major validation studies, performed in samples of human origin, the overall specificity of the assay was found to be >97% [19,21,22], while the overall reproducibility and negative percent agreement are considered to be >99% [23].

In samples of veterinary interest, the test has been employed infrequently. The technology was first employed by Rodriguez et al. [24] in samples from hedgehogs in Spain. That work was followed by a study from our group in samples from sheep and goats [25].

There is interest in monitoring the gastrointestinal pathogens with zoonotic potential in horses because of the closeness of these animals with people. The objectives of this study were (a) to detect gastrointestinal pathogens with a zoonotic potential in faecal samples of horses using the FilmArray^®^ GI Panel and (b) to identify variables potentially associated with the presence of the potentially zoonotic agents in equine faecal samples.

## 2. Materials and Methods

### 2.1. Animals and Samplings

**Figure 1:**
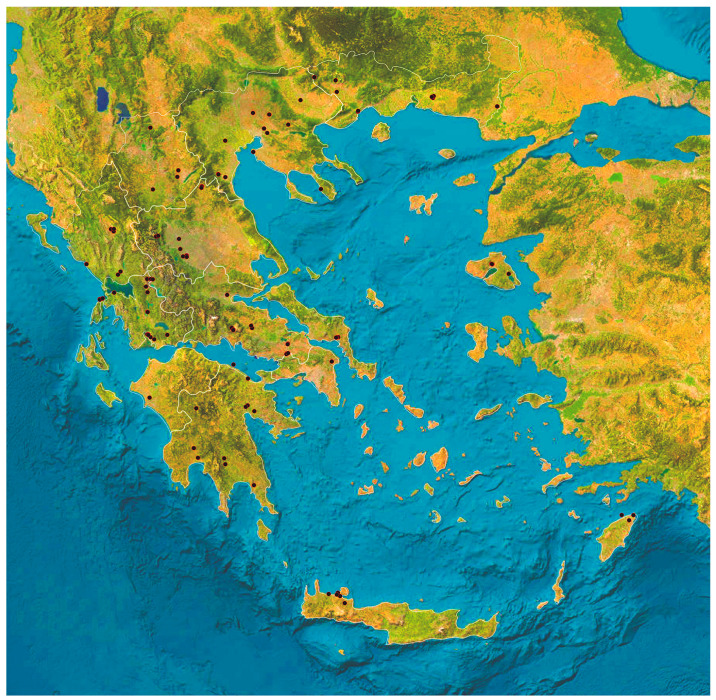
Location of horses (*n* = 224) from which faecal samples were collected in Greece.

This cross-sectional study was performed from March 2023 to March 2024 in a countrywide study across the 13 administrative regions of Greece. In total, 224 horses were included in the study and sampled. A map of the locations of horses from which samples were obtained is in Figure 1.

The inclusion criteria for animals in the study were (a) horse age (≥1 year), (b) good clinical condition of the horse, and (c) animal’s sole involvement in free-riding activities. The exclusion criteria were (a) administration of antibiotics during the six months prior to sampling and (b) animal’s housing in equine club facilities and its participation in organized equestrian activities. Thereafter, animals were included in the study on a convenience basis, i.e., the willingness of horse owners to accept sampling of their animal from university staff.

Before sample collection, a detailed clinical examination of the horse was performed to confirm absence of clinically evident abnormalities; special attention was paid to absence of clinical signs potentially associated with gastrointestinal infections (e.g., diarrhoea). Information regarding parameters related to the living conditions of horses was obtained by means of a short questionnaire (Table S1).

Faecal samples were collected directly into the gloved hand of the investigator, as these were excreted from the rectum of the animals. Approximately 30 g of individual animal faecal samples were taken and homogenized in phosphate-buffered saline by mild bead-beating [26]. Finally, swab samples were taken from the homogenized samples and immersed into Cary Blair transport media.

Swab samples were stored at 1.0 to 4.0 °C using portable refrigerators. Transportation of samples to the laboratory was carried out by the investigators.

### 2.2. Laboratory Examinations

Laboratory examinations started within 24 h after collection of samples. All the faecal swab samples were tested by means of the BioFire^®^ FilmArray^®^ Gastrointestinal (GI) Panel (BioFire Diagnostics, Salt Lake City, UT, USA). The panel uses a two-stage nested multiplex PCR process and, specifically for RNA viruses, reverse transcription [27]. It has the capacity to detect genetic material of the following microorganisms: (i) *Campylobacter* (*jejuni*, *coli*, *upsaliensis*), (ii) *Clostridium difficile* (toxin A/B), (iii) *Plesiomonas shigelloides*, (iv) *Salmonella* spp., (v) *Vibrio* (*parahaemolyticus*, *vulnificus*, *cholerae*), (vi) *Vibrio cholerae*, (vii) *Yersinia enterocolitica*, (viii) enteroaggregative *Escherichia coli*, (ix) enteropathogenic *E. coli*, (x) enterotoxigenic *E. coli* lt/st, (xi) Shiga-like toxin-producing *E. coli* stx1/stx2, (xii) *E. coli* O157, (xiii) *Shigella*/entero-invasive *E. coli*, (xiv) *Cryptosporidium*, (xv) *Cyclospora cayetanensis*, (xvi) *Entamoeba histolytica*, (xvii) *Giardia duodenalis*, (xviii) Adenovirus F40/41, (xix) Astrovirus, (xx) Norovirus GI/GII, (xxi) Rotavirus A and (xxii) Sapovirus (I, II, IV, and V).

Each swab was immersed initially into 500 μL trypticase soya broth. Then, a quantity of 200 μL was added to the panel according to the manufacturer’s instructions [27], for analysis in The BioFire^®^ FilmArray^®^ 2.0 System (BioFire Diagnostics, Salt Lake City, UT, USA). The actual test was completed within one hour.

### 2.3. Data Management and Analysis

Data were entered into Microsoft Excel (version 2407 *(Build 17830.20138)*) and analyzed using SPSS v. 21 (IBM Analyt-ics, Armonk, NY, USA). A basic descriptive analysis was performed, and exact binomial confidence intervals (CIs) were obtained.

Initially, the outcomes of ‘detection of at least one zoonotic pathogen in the faecal sample from an animal’ and ‘detection of two zoonotic pathogens concurrently in the faecal sample from an animal’ were considered. Subsequently, the outcomes ‘detection of a specific zoonotic pathogen (xxx) in the faecal sample from an animal’ (where xxx: *Y. enterocolitica*, virulent *E. coli*, *G. duodenalis* or Norovirus GI/GII, i.e., in total four different outcomes) were considered. Variables related to sampling conditions, to the characteristics of the horse and to the management and living conditions of the animal were evaluated for potential association with the presence of pathogens in faecal samples (Table S1). For each of these variables, categories were created. The importance of predictors was assessed in univariable analysis by means of the Pearson’s chi-square test or the Mann–Whitney test, as appropriate.

Thereafter, a multivariable model was developed for each of the above outcomes; parameters found with *p* < 0.2 in the preceding univariable analyses were offered to the respective model. Progressively, variables into a multivariable model were removed from that by using backwards elimination. The likelihood ratio test was performed to assess the *p*-value of each parameter; among those found with *p* ≥ 0.2, the one with the largest *p* was removed from the model. The procedure was repeated until no variable with *p* ≥ 0.2 could be removed from the model. The variables included in the final multivariable models constructed are detailed in Table S2.

Finally, for the outcome ‘detection of at least one zoonotic pathogen in the faecal sample from an animal’, variables found to be significant in the univariable analysis were subsequently included in principal component analysis.

In all analyses, statistical significance was defined at *p* < 0.05.

## 3. Results

### 3.1. Detection of Potentially Zoonotic Pathogens in Faecal Samples

Zoonotic pathogens were detected in the faecal samples obtained from 97 horses (43.3% (95% confidence interval (CI): 37.0–49.9%)). The median number of pathogens detected was 0 (interquartile range: 1) per horse.

**Figure 2:**
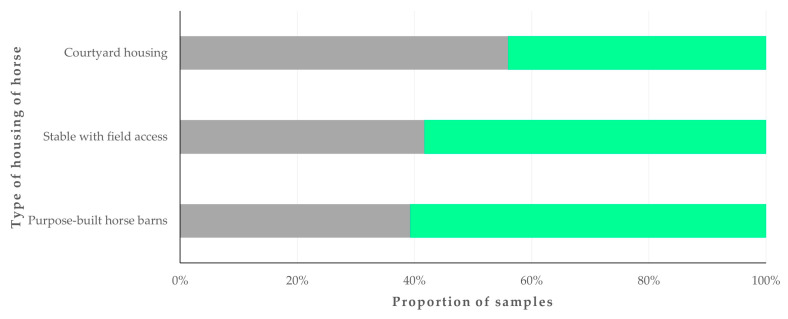
Proportion of faecal samples from horses (*n* = 224) in Greece, in which zoonotic gastrointestinal pathogens were detected, in accordance with the type of horse housing. Grey bars: proportions of horses in which pathogens were detected; green bars: proportions of horses in which pathogens were not detected.

Zoonotic pathogens were detected more frequently in samples from horses in courtyard housing (56.0%) than in samples from horses in other housing types (39.7%) (*p* = 0.040) (Figure 2).

The most frequently detected pathogens were enteropathogenic *E. coli*, from 43 animals (19.2% (95% CI: 14.6–24.9%)), and Shiga-like toxin-producing *E. coli* stx1/stx2 from 31 animals (13.8% (95% CI: 9.9–19.0%)). Other pathogens detected were enterotoxigenic *E. coli* lt/st, *E. coli* O157, *Giardia duodenalis* (also named *G. intestinalis* or *G. lamblia*) *Yersinia enterocolitica*, *Shigella*/enteroinvasive *E. coli*, Norovirus GI/GII, *Clostridium difficile* (toxin A/B), *Salmonella* spp. and *Vibrio* spp. (Table 1).

### 3.2. Predictors

**Figure 3:**
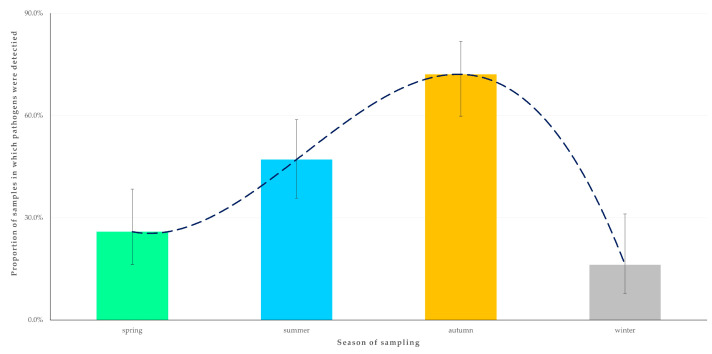
Seasonal change in the detection rate of zoonotic gastrointestinal pathogens in faecal samples from horses (*n* = 224) in Greece. The dashed line is the trendline.

In the univariable analysis for the outcome ‘detection of at least one zoonotic pathogen in the faecal sample from an animal’, season when sampling took place (Figure 3), age of horse, and presence of livestock (cattle, sheep, goats or pigs) at the same premises as horses were found to be significant (Table S3).

**Figure 4:**
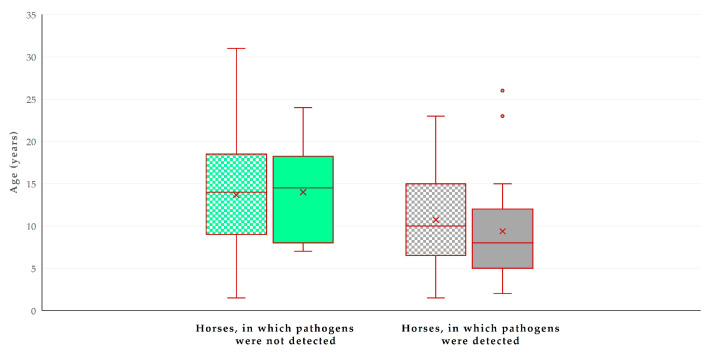
Box and whisker plot of the age of horses (*n* = 224) in Greece, in accordance with the detection of zoonotic gastrointestinal pathogens in faecal samples and the presence of livestock at the same premises. Green bars: horses in which pathogens were not detected; grey bars: horses in which pathogens were detected; motif pattern: no presence of livestock at same premises; full pattern: presence of livestock at same premises.

**Figure 5:**
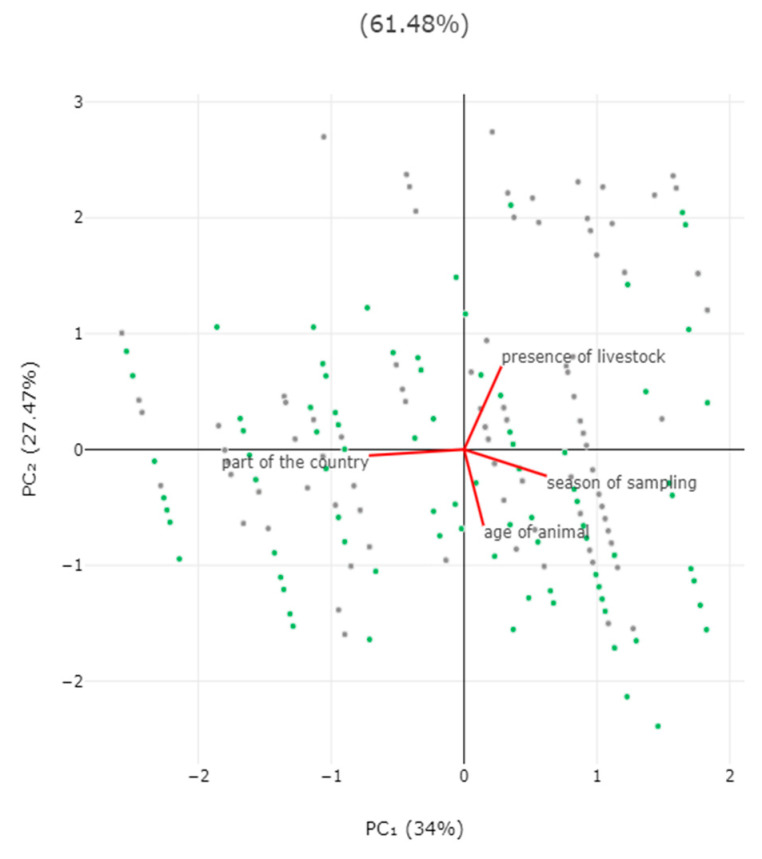
Biplot of results of principal component analysis for detection of zoonotic gastrointestinal pathogens in faecal samples from horses in Greece, in accordance with season when sampling took place, location of horse (part of the country), age of horse, and presence of livestock at the same premises. Grey dots: horses in which pathogens were detected; green dots: horses in which pathogens were not detected.

In the multivariable analysis, the following two variables emerged as significant predictors for the detection of zoonotic pathogens in faecal samples: (a) decreasing age of the horse (*p* = 0.0001) and (b) presence of livestock at the same premises (*p* = 0.013) (Table 3, Figure 4). The principal component analysis revealed that the two principal components accounted for 61.5% of the variation between horses (Figure 5 and Figure S1, Table S4).

**Figure 6:**
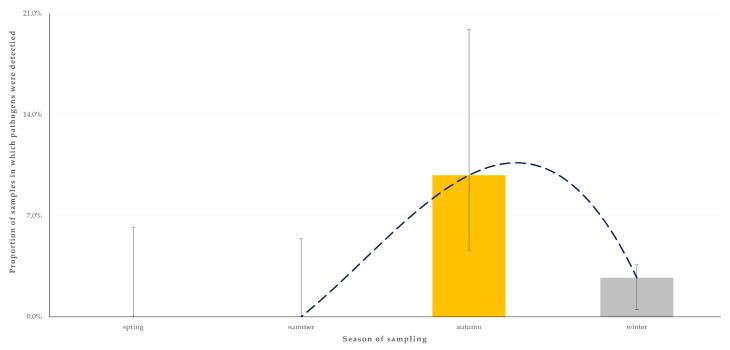
Seasonal change in the detection rate of two zoonotic gastrointestinal pathogens concurrently in faecal samples from horses (*n* = 224) in Greece. The dashed line is the trendline.

For the outcome ‘detection of two zoonotic pathogens concurrently in the faecal sample from an animal’, only the season when sampling took place (*p* = 0.049) emerged as a significant predictor after the univariable (Table S5) and the multivariable analysis (Table 4). Most samples in which two pathogens were detected (83.3%) were obtained during the autumn (Figure 6).

The results of the analyses for specific pathogens (*Y. enterocolitica* and virulent *E. coli*) also revealed the age of the horse, the presence of livestock at the same premises, and the season when sampling took place as significant predictors of the detection of these two pathogens (Table 5 and Table 6). For the detection of *G. duodenalis*, the location of horses on the islands of the country was found to have a significant association in the univariable analysis, but this was not confirmed in the multivariable evaluation (*p* = 0.31). For the detection of Norovirus GI/GII, no significant predictors emerged (*p* > 0.37).

## 4. Discussion

### 4.1. Frequency of Detection and Identity of Potentially Zoonotic Pathogens

The gastrointestinal tract of animals harbours a complex and dynamic microbial population, which plays roles in digestion, metabolism and immunomodulation [28]. This study presents an extended and countrywide investigation of pathogens with zoonotic potential in horses in Greece, where, notably, this is the largest ever field investigation performed in that animal species. The inclusion of animals from various housing settings at locations across all administrative regions of Greece provides an overview of the conditions prevailing throughout the country and encompasses a variety of local conditions. In this respect, the study is also among the largest internationally.

This study included horses active in free-riding activities rather than ones in racecourses, as the former animals live in closer contact with humans, hence these would potentially pose a comparatively higher risk for pathogen transmission to people. Horse owners may come in close contact with their animals in various ways, particularly during handling, grooming, riding or petting them [29]. Overall, the findings confirmed that a proportion of clinically healthy horses harboured pathogens with zoonotic potential, which can possibly be transferred, through various routes, to humans who may come in contact with their animals.

Although faecal samples were homogenized before processing, differences in faecal consistency could have led to some variations in the results [30]; for example, pathogens might have concentrated at specific areas in faecal balls as the result of localization of bacteria within the intestine or as the consequence of the moisture content of faecal material [31]. To a large extent, sample preparation by bead-beating contributed to minimizing such effects; however, there is still a possibility that some pathogens might have not been detected, contributing to some underestimate of the prevalence rate of pathogen presence.

Detection was more frequent among animals housed at the location of owners’ home, which, firstly, indicates a comparatively higher risk for pathogen transmission: one may reasonably assume that when animals are living at the courtyard of their owners’ house, people come into contact with the animals more frequently. Further, it is also possible that the increased detection may possibly be the result of pathogen transmission from people to horses, also as the consequence of the frequent contact between the two hosts [32,33,34]. Nevertheless, human–equine interactions are interrelated and complex and should be interpreted with care [35]. Nevertheless, further potential sources of pathogens can exist, as discussed below.

In general, the various pathogens detected during this study are those most frequently reported in previous studies in faecal samples from healthy horses [36,37,38]. A significant exception was Norovirus, the detection of which in samples from horses had not been reported previously. Possibly, one may postulate that the pathogen might have been transferred to the animals from the owners or animal handlers, although infection from watering sources contaminated with the pathogen cannot be excluded [39].

Among the various pathogens detected, there is early (1978) evidence of infection of people with *Salmonella* (specifically, *S. typhimurium* var. copenhagen) from horses [40]. Apart from the potential of *Salmonella* to infect people through direct contact, e.g., in riding clubs or in veterinary hospitals [41], one should also take into account the largely ignored possibility of *Salmonella* transmission from equine carcasses, particularly in areas where consumption of raw equine meat is practiced [42].

The higher frequency of detection of *E. coli* and *Y. enterocolitica* among the pathogens can be attributed overall to their robust virulence factors, environmental resilience, and strong ability to colonize equine hosts. For *E. coli*, these include toxins disrupting host’s cellular processes, adhesins facilitating bacterial attachment on equine gut epithelial cells, and secretion systems supporting bacterial evasion of horse immune defences [43,44]. The high number of serotypes among *E. coli* further contributes to the high frequency of isolation of this organism. For *Y. enterocolitica*, these refer to the easy adaptation to various tissue environments, the effective countering of immune defences through phagocytosis inhibition, and survival in a variety of environmental conditions [45,46]. Cumulatively, the above could have possibly contributed to the higher detection rates of these bacteria compared with the other pathogens.

### 4.2. The Use of FilmArray^®^ GI Panel Technology in Samples from Horses

FilmArray^®^ GI Panel uses multiplex–PCR technology with the aim of detecting the most common enteropathogenic organisms in humans and has the advantage that the assay can be employed directly in faecal samples. The time to completion of the assay is approximately one hour, which contributes to a rapid diagnosis.

The technology has not been reported to have been used previously on samples collected from horses and has only been used twice on animal samples. Only Rodriguez et al. [24] and Tsilipounidaki et al. [25] have reported use of the assay in samples from hedgehogs and lambs and kids, respectively. The method was found to be useful and convenient. Further, by using this assay, it was possible to detect pathogens such as Norovirus, which would not normally be diagnosed by using standard equine-oriented laboratory methodologies.

With regard to the comparison of the results of the technology against those of conventional faecal cultures and molecular methods, reference is made to the extensive study of Buss et al. [19], who assessed 1556 faecal samples and compared the results of the two approaches. These authors reported that the sensitivity of FilmArray^®^ GI Panel was 100% for *P. shigelloides*, *Salmonella* spp., *Y. enterocolitica*, enterotoxigenic *E. coli* lt/st, *E. coli* O157, *Cryptosporidium* spp., *C. cayetanensis*, *G. lamblia*, Astrovirus, Rotavirus A and Sapovirus and 95% for *Campylobacter* spp., *C. difficile* (toxin A/B), enteroaggregative *E. coli*, enteropathogenic *E. coli*, *Shigella*/enteroinvasive *E. coli*, Adenovirus F 40/41, and Norovirus.

Hence, there is potential for this technology to be used in samples of equine origin for the specific detection of pathogens of zoonotic importance. Indeed, the ease of use and the quick reporting of results further support its use.

The main limitation of the assay refers to the detection of genetic material rather than pathogens present in the gastrointestinal tract at the time of sampling (i.e., active infections). This may account partly for a discrepancy reported in individuals with disease between the clinical diagnosis and the results of the assay [47].

### 4.3. Predictors

Younger animals, found to be associated with more frequent pathogen detection, may need time to develop a mature immune system of the gastrointestinal tract. Moreover, younger horses may have a higher susceptibility to infections, possibly due to their developing immune systems [48,49]. In general, it may be more likely for younger animals to develop a bacterial shedding state [50], a fact shown for the faecal excretion of *E. coli* [51], a specific pathogen for the detection of which the young age of horses emerged as a predictor in the present study.

Further, microbial populations in an animal environment depend on the animals present there, as well as on additional factors: people, plants, soil, feeds, water (as can be the case with Norovirus [39]), and fomites. In this respect, herbivore animals uptake and disseminate pathogens during grazing or browsing. Herbage, drinking water, and fomites contaminated with pathogens with zoonotic potential of equine origin represent a significant public health concern, as well as possible sources of infection of horses themselves [52]. Horses may act as reservoirs for various pathogens, e.g., *Salmonella*, which can contaminate their environment through shedding in faeces. In this way, pathogens can spread to herbage, drinking water, and fomites (e.g., stable equipment, grooming tools), posing a risk to people [53,54].

Studies on the intestinal microbiome have shown similarities between horses and goats [55] and horses and cattle [56]. Further, Park and Kim [56] reported that, for most bacterial phyla evaluated, populations in cattle were found to be higher than in horses, whilst Pusterla et al. [57] reported that exposure to pig faeces can be a potential source of gastrointestinal pathogen infection for horses. Cohabitation with livestock indicates a possible cross-species transmission or, alternatively, a shared environmental contamination. These studies support a hypothesis of pathogen dissemination from livestock to horses, which is compatible with the findings of our predictor analysis.

Overall, the present findings are consistent with previous studies that reported that young animals are more prone to infections and that mixed-species farming can facilitate pathogen spread [37].

Additionally, the season of sampling (autumn) emerged as a significant predictor of the detection of two zoonotic pathogens concurrently. Seasonal variations in pathogen prevalence have been reported in previous studies, likely as a result of changes in environmental conditions, animal behaviour, and management practices at different time-points throughout a year [58,59]. These seasonal changes could guide strategic timing for interventions and monitoring efforts to mitigate pathogen spread.

## 5. Conclusions

The added value of this study is the detection of gastrointestinal pathogens with zoonotic potential in horses; that proportion was higher among those living in house courtyard facilities. A diversity of pathogens—viruses, bacteria, protozoa—was detected. *E. coli* was the primary pathogen found. The detection of pathogens with zoonotic potential in samples collected from healthy animals indicates that people should be cautious during contact with these animals. Moreover, precautionary measures (e.g., personal hygiene) should be maintained during and after the contact. The results also provided evidence for predictors for the infections; they showed that the younger age of horses, the presence of livestock with the horses, and the season of sampling were significant predictors.

The results of the study can serve to inform horse owners and handlers regarding the possible risk of transmission of pathogens with zoonotic potential from these animals. In addition, the findings highlight the importance of continuous surveillance for zoonotic pathogens in domestic animals.

## Figures and Tables

**Table 1:** Detection of zoonotic gastrointestinal pathogens in faecal samples from horses (*n* = 97) in Greece.

Pathogens	Frequency of Detection (*n*) (Prevalence (95% CI))
*Campylobacter* spp. (*jejuni*, *coli*, *upsaliensis*)	0 (0.0%, 0.0–1.7%)
*Clostridium difficile* (toxin A/B)	1 (0.5%, 0.1–2.5%)
*Plesiomonas shigelloides*	0 (0.0%, 0.0–1.7%)
*Salmonella* spp.	1 (0.5%, 0.1–2.5%)
*Vibrio* spp. (*parahaemolyticus*, *vulnificus*, *cholerae*)	1 (0.5%, 0.1–2.5%)
*Vibrio cholerae*	0 (0.0%, 0.0–1.7%)
*Yersinia enterocolitica*	7 (3.1%, 1.5–6.3%)
Enteroaggregative *Escherichia coli*	0 (0.0%, 0.0–1.7%)
Enteropathogenic *E. coli*	43 (19.2%, 14.6–24.9%)
Enterotoxigenic *E. coli* lt/st	16 (7.1%, 4.4–11.3%)
Shiga-like toxin-producing *E. coli* stx1/stx2	31 (13.8%, 9.9–19.0%)
*E. coli* O157	11 (4.9%, 2.8–8.6%)
*Shigella*/enteroinvasive *E. coli*	2 (0.9%, 0.2–3.5%)
*Cryptosporidium* spp.	0 (0.0%, 0.0–1.7%)
*Cyclospora cayetanensis*	0 (0.0%, 0.0–1.7%)
*Entamoeba histolytica*	0 (0.0%, 0.0–1.7%)
*Giardia duodenalis*	7 (3.1%, 1.5–6.3%)
Adenovirus F40/41	0 (0.0%, 0.0–1.7%)
Astrovirus	0 (0.0%, 0.0–1.7%)
Norovirus GI/GII	2 (0.9%, 0.2–3.5%)
Rotavirus A	0 (0.0%, 0.0–1.7%)
Sapovirus (I, II, IV, and V)	0 (0.0%, 0.0–1.7%)

Two pathogens concurrently were detected in six horses (2.7% (95% CI: 1.2–5.7%)). The most commonly detected combination of different pathogens was enteropathogenic *E. coli* with *G. duodenalis* (three horses) (Table 2).

**Table 2:** Combinations of zoonotic gastrointestinal pathogens detected in faecal samples from horses (*n* = 6) in Greece.

Combinations of Pathogens	Frequency of Detection (*n*)
Enteropathogenic *E. coli* and *G. duodenalis*	3
Enteropathogenic *E. coli* and *Salmonella*	1
Enterotoxigenic *E. coli* lt/st—Shiga-like toxin-producing *E. coli* stx1/stx2—*E. coli* O157 and Norovirus GI/GII	1
Shiga-like toxin-producing *E. coli* stx1/stx2—*E. coli* O157 and Norovirus GI/GII	1

**Table 3:** Results of multivariable analysis for the detection of zoonotic gastrointestinal pathogens in faecal samples from horses (*n* = 224) in Greece.

Variables	Odds Risk/Odds Ratio ^1^	*p*-Value
Age of horse		0.0001
Per unit (year) decrease	1.021 ± 1.005	<0.0001
Presence of livestock at the same premisesas horses		0.014
Yes (63.2% ^2^)	2.654 (1.289–5.462)	0.008
No (39.2%)	reference	

^1^ ± standard error/(95% confidence interval); ^2^ proportion of samples in which zoonotic pathogens were detected.

**Table 4:** Results of multivariable analysis for the detection of two zoonotic gastrointestinal pathogens concurrently in faecal samples from horses (*n* = 224) in Greece.

Variables	Odds Ratios ^1^	*p*-Value
Season when sampling of horse took place		0.049
Spring (0.0% ^2^)	1.171 (0.023–59.938)	0.937
Summer (0.0%)	reference	
Autumn (8.2%)	13.336 (0.722–246.398)	0.08
Winter (2.7%)	5.630 (0.224–141.727)	0.29

^1^ (95% confidence interval); ^2^ proportion of samples in which two zoonotic pathogens were detected.

**Table 5:** Multivariable analysis for variables significantly associated with the detection of *Y. enterocolitica* in faecal samples from horses (*n* = 224) in Greece.

Variables	Odds Ratios/Odds Risk ^1^	*p*-Value
Presence of livestock at the same premises		0.004
Yes (57.1% ^2^)	7.177 (1.537–33.511)	0.012
No (15.7%)	reference	

^1^ (95% confidence interval)/± standard error; ^2^ proportion of samples in which zoonotic pathogens were detected.

**Table 6:** Multivariable analysis for variables significantly associated with the detection of virulent *E. coli* in faecal samples from horses (*n* = 224) in Greece.

Variables	Odds Ratios/Odds Risk ^1^	*p*-Value
Age of horse		0.0006
Per year decrease	1.017 ± 1.005	0.0004
Presence of livestock at the same premises		0.032
Yes (24.7% ^2^)	2.277 (1.123–4.615)	0.023
No (12.6%)	reference	
Season when sampling took place		0.033
Spring (17.2%)	1.333 (0.417–4.266)	0.63
Summer (35.3%)	3.491 (1.203–10.134)	0.022
Autumn (68.9%)	14.147 (4.770–41.961)	<0.0001
Winter (13.5%)	reference	

^1^ (95% confidence interval)/± standard error; ^2^ proportion of samples in which zoonotic pathogens were detected.

## Data Availability

All data associated with this research are available in supplementary material.

## Supplementary Materials

The following supporting information can be downloaded at: https://www.mdpi.com/article/10.3390/ani14172566/s1, Table S1: Details recorded during a countrywide investigation for gastrointestinal zoonotic pathogens in 224 horses in Greece; Table S2: Details of multivariable models (*n* = 6) employed for the evaluation of associations with the detection of zoonotic pathogens in faecal samples from 224 horses during a countrywide study in Greece; Table S3: Results of univariable analysis for predictors for detection of at least one zoonotic gastrointestinal pathogen in faecal samples from 224 horses during a countrywide study in Greece; Table S4: Eigenvalues for principal component analysis for detection of zoonotic pathogens in faecal samples from horses, in accordance with season of sampling, part of the country, age of horses and presence of livestock at the same premises; Figure S1: Scree-plot of results of principal components analysis for detection of zoonotic pathogens in faecal samples from horses, in accordance with season of sampling, part of the country, age of horses and presence of livestock at the same premises; Table S5: Results of univariable analysis for predictors for detection of two zoonotic gastrointestinal pathogens concurrently in faecal samples from 224 horses during a countrywide study in Greece.
