# Supplementary material for: Detection of Gastrointestinal Pathogens with Zoonotic Potential in Horses Used in Free-Riding Activities during a Countrywide Study in Greece
Source: Animals (Basel). 2024 Sep 3;14(17):2566. doi: 10.3390/ani14172566 (PMC11394066; doi:10.3390/ani14172566)
Supplement: Supplementary file 1 [file animals-14-02566-s001.zip › animals-3173651-supplementary.pdf]

Detection of Gastrointestinal Pathogens with Zoonotic Potential in Horses Used in Free-Riding Activities During a Countrywide Study in Greece

P. Tyrnenopoulou, K. Tsilipounidaki, Z. Florou, C.-G. Gkountinoudis, K. Tyropoli, A. Starras, C. Peleki, D. Marneris, N. Arseniou, D.T. Lianou, E.I. Katsarou, E. Petinaki, and G.C. Fthenakis

Table S1. Details recorded during a countrywide investigation for gastrointestinal zoonotic pathogens in 224 horses in Greece.

Season when sampling of horse took place (description)
Location of horse (part of the country) (description)
Gender of horse (male / female)
Age of horse (years)
Type of housing of horse (horse stable, barn and field, courtyard housing)
Anthelmintic administration to horse during the last two months prior to sampling (yes / no)
Vaccination of horse against influenza / tetanus during the last year prior to sampling (yes / no)
Access of horse to an open field (yes / no)
Presence of livestock at the same premises with horse (yes / no)
Presence of domestic companion animals at the same premises with horse (yes / no)
Presence of poultry at the same premises with horse (yes / no)
Presence of South American camelids at the same premises with horse (yes / no)

Table S2. Details of multivariable models (n = 6) employed for the evaluation of associations with the detection of zoonotic pathogens in faecal samples from 224 horses during a countrywide study in Greece.

Outcome	Variables		
	**assessed in univariable analyses (*n*)**	**offered to the multivariable models (*n*)**	**required in the final models**
Detection of at least one zoonotic pathogen in the faecal sample from an animal	12	5	(a) age of horse, (b) presence of livestock at the same premises with horse
Detection of two zoonotic pathogens in the faecal sample from an animal	12	1	(a) season when sampling of horse took place
Detection of *Y. enterocolitica* in the faecal sample from an animal	12	2	(a) presence of livestock at the same premises with horse
Detection of virulent *E. coli* in the faecal sample from an animal	12	5	(a) season when sampling of horse took place, (b) age of horse, (c) presence of livestock at the same premises with horse
Detection of *G. duodenalis* in the faecal sample from an animal	12	1	(a) location of horse (part of the country)
Detection of Norovirus GI / GII in the faecal sample from an animal	12	1	(a) season when sampling of horse took place

Table S3. Results of univariable analysis for predictors for detection of at least one zoonotic gastrointestinal pathogen in faecal samples from 224 horses during a countrywide study in Greece.

Horses from which no zoonotic gastrointestinal pathogens were detected (n = 127)				Horses from which zoonotic gastrointestinal pathogens were detected (n = 97)				p
**Season when sampling of horse took place**								
Spring	Summer	Autumn	Winter	Spring	Summer	Autumn	Winter	
43	36	17	31	15	32	44	6	< 0.0001
**Location of horse (part of the country)**								
North part	Central part	South part	Islands	North part	Central part	South part	Islands	
42	46	29	10	38	32	11	16	0.040
**Gender of animal**								
Female	Male			Female	Male			
59	68			51	46			0.36
**Age of horse**								
median: 14.0 (9.0) years				median: 10.0 (8.0) years				< 0.0001
**Type of housing of horse**								
Purpose-built horse barns	Stable with field access	Courtyard housing		Purpose-built horse barns	Stable with field access	Courtyard housing		
91	14	22		59	10	28		0.12
**Anthelmintic administration to horse during the last two months prior to sampling**								
Yes	No			Yes	No			
84	43			64	33			0.98
**Vaccination of horse against influenza / tetanus during the last year prior to sampling**								
Yes	No			Yes	No			
81	46			55	42			0.28
**Access of horse to an open field**								
Yes	No			Yes	No			
53	74			43	54			0.70
**Presence of livestock at the same premises with horse**								
Yes	No			Yes	No			
14	113			24	73			0.007
**Presence of domestic companion animals at the same premises with horse**								
Yes	No			Yes	No			
90	37			67	30			0.77

Presence of poultry at the same premises with horse				
Yes	No	Yes	No	
10	117	6	91	0.63
Presence of South American camelids at the same premises with horse				
Yes	No	Yes	No	
3	124	3	94	0.74

Table S4. Eigenvalues for principal component analysis for detection of zoonotic pathogens in faecal samples from horses, in accordance with season of sampling, part of the country, age of horses and presence of livestock at the same premises.

Parameter	Component			
	PC_1	PC_2	PC_3	PC_4
Eigenvalue	1.36	1.10	0.93	0.61
% of Variance	34.0	27.5	23.2	15.4
Cumulative variance	34.0%	61.5%	84.6%	100.0%

Figure S1. Scree-plot of results of principal components analysis for detection of zoonotic pathogens in faecal samples from horses, in accordance with season of sampling, part of the country, age of horses and presence of livestock at the same premises. The dashed lines are the trendlines.

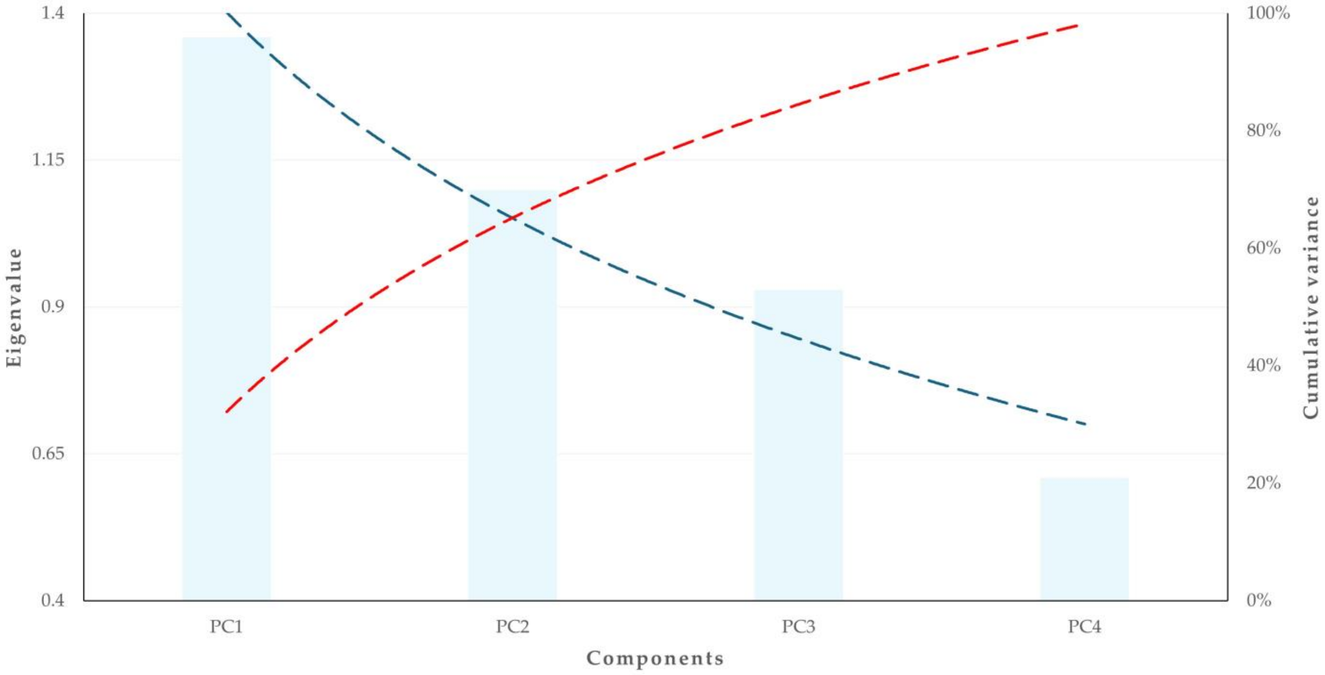

Table S5. Results of univariable analysis for predictors for detection of two zoonotic gastrointestinal pathogens concurrently in faecal samples from 224 horses during a countrywide study in Greece.

<table>
<tr><th colspan="12">Horses from which less than two zoonotic gastro-intestinal pathogens were detected (n = 118)</th><th colspan="12">Horses from which two zoonotic gastro-intestinal pathogens were detected (n = 6)</th><th>p</th></tr>
<tr><td colspan="25">Season when sampling of horse took place</td></tr>
<tr><td colspan="3">Spring</td><td colspan="3">Summer</td><td colspan="3">Autumn</td><td colspan="3">Winter</td><td colspan="3">Spring</td><td colspan="3">Summer</td><td colspan="3">Autumn</td><td colspan="3">Winter</td><td></td></tr>
<tr><td colspan="3">58</td><td colspan="3">68</td><td colspan="3">56</td><td colspan="3">36</td><td colspan="3">0</td><td colspan="3">0</td><td colspan="3">5</td><td colspan="3">1</td><td>0.014</td></tr>
<tr><td colspan="25">Location of horse (part of the country)</td></tr>
<tr><td colspan="3">North part</td><td colspan="3">Central part</td><td colspan="3">South part</td><td colspan="3">Islands</td><td colspan="3">North part</td><td colspan="3">Central part</td><td colspan="3">South part</td><td colspan="3">Islands</td><td></td></tr>
<tr><td colspan="3">78</td><td colspan="3">75</td><td colspan="3">40</td><td colspan="3">25</td><td colspan="3">2</td><td colspan="3">3</td><td colspan="3">0</td><td colspan="3">1</td><td>0.65</td></tr>
<tr><td colspan="25">Gender of horse</td></tr>
<tr><td colspan="6">Female</td><td colspan="6">Male</td><td colspan="6">Female</td><td colspan="6">Male</td><td></td></tr>
<tr><td colspan="6">107</td><td colspan="6">111</td><td colspan="6">3</td><td colspan="6">3</td><td>0.96</td></tr>
<tr><td colspan="25">Age of horse</td></tr>
<tr><td colspan="12">median: 12.0 (10.0) years</td><td colspan="12">median: 11.5 (12.0) years</td><td>0.88</td></tr>
<tr><td colspan="25">Type of housing of horse</td></tr>
<tr><td colspan="4">Horse stable</td><td colspan="4">Barn and field</td><td colspan="4">Courtyard housing</td><td colspan="4">Horse stable</td><td colspan="4">Barn and field</td><td colspan="4">Courtyard housing</td><td></td></tr>
<tr><td colspan="4">145</td><td colspan="4">24</td><td colspan="4">49</td><td colspan="4">5</td><td colspan="4">0</td><td colspan="4">1</td><td>0.61</td></tr>
<tr><td colspan="25">Anthelmintic administration to horse during the last two months prior to sampling</td></tr>
<tr><td colspan="6">Yes</td><td colspan="6">No</td><td colspan="6">Yes</td><td colspan="6">No</td><td></td></tr>
<tr><td colspan="6">145</td><td colspan="6">73</td><td colspan="6">3</td><td colspan="6">3</td><td>0.40</td></tr>
<tr><td colspan="25">Vaccination of horse against influenza / tetanus during the last year prior to sampling</td></tr>
<tr><td colspan="6">Yes</td><td colspan="6">No</td><td colspan="6">Yes</td><td colspan="6">No</td><td></td></tr>
<tr><td colspan="6">133</td><td colspan="6">85</td><td colspan="6">3</td><td colspan="6">3</td><td>0.59</td></tr>
<tr><td colspan="25">Access of horse to an open field</td></tr>
<tr><td colspan="6">Yes</td><td colspan="6">No</td><td colspan="6">Yes</td><td colspan="6">No</td><td></td></tr>
<tr><td colspan="6">93</td><td colspan="6">125</td><td colspan="6">3</td><td colspan="6">3</td><td>0.72</td></tr>
<tr><td colspan="25">Presence of livestock at the same premises with horse</td></tr>
<tr><td colspan="6">Yes</td><td colspan="6">No</td><td colspan="6">Yes</td><td colspan="6">No</td><td></td></tr>
<tr><td colspan="6">38</td><td colspan="6">180</td><td colspan="6">0</td><td colspan="6">6</td><td>0.26</td></tr>
<tr><td colspan="25">Presence of domestic companion animals at the same premises with horse</td></tr>
<tr><td colspan="6">Yes</td><td colspan="6">No</td><td colspan="6">Yes</td><td colspan="6">No</td><td></td></tr>
<tr><td colspan="6">152</td><td colspan="6">66</td><td colspan="6">5</td><td colspan="6">1</td><td>0.47</td></tr>
</table>

Presence of poultry at the same premises with horse				
Yes	No	Yes	No	
16	202	0	6	0.49
Presence of South American camelids at the same premises with horse				
Yes	No	Yes	No	
6	212	0	6	0.68
